# The Affordable Medicines Facility-malaria (AMFm): are remote areas benefiting from the intervention?

**DOI:** 10.1186/s12936-015-0904-z

**Published:** 2015-10-09

**Authors:** Yazoume Ye, Fred Arnold, Abdisalan Noor, Marilyn Wamukoya, John Amuasi, Samuel Blay, Blessing Mberu, Ruilin Ren, Catherine Kyobutungi, Frederick Wekesah, Hellen Gatakaa, Mitsuru Toda, Julius Njogu, Illah Evance, Kathryn O’Connell, Tanya Shewchuk, Sarah Thougher, Andrea Mann, Barbara Willey, Catherine Goodman, Kara Hanson

**Affiliations:** ICF International, 530 Gaither Road, Suite 500, Rockville, MD 20850 USA; KEMRI/Wellcome Trust, Nairobi, Kenya; African Population and Health Research Center, Nairobi, Kenya; Kumasi Centre for Collaborative Research in Tropical Medicine, Kumasi, Ghana; Komfo Anokye Teaching Hospital, Kumasi, Ghana; Institute of Tropical Medicine (NEKKEN), Nagasaki University, Nagasaki, Japan; The ACTwatch project (Population Services International), Nairobi, Kenya; Department of Global Health and Development, London School of Hygiene and Tropical Medicine, London, UK

**Keywords:** Remote areas, Non-remote areas, Access to malaria treatment, Availability of ACT, Quality-assured ACT

## Abstract

**Background:**

To assess the availability, price and market share of quality-assured artemisinin-based combination therapy (QAACT) in remote areas (RAs) compared with non-remote areas (nRAs) in Kenya and Ghana at end-line of the Affordable Medicines Facility-malaria (AMFm) intervention.

**Methods:**

Areas were classified by remoteness using a composite index computed from estimated travel times to three levels of service centres. The index was used to five categories of remoteness, which were then grouped into two categories of remote and non-remote areas. The number of public or private outlets with the potential to sell or distribute anti-malarial medicines, screened in nRAs and RAs, respectively, was 501 and 194 in Ghana and 9980 and 2353 in Kenya. The analysis compares RAs with nRAs in terms of availability, price and market share of QAACT in each country.

**Results:**

QAACT were similarly available in RAs as nRAs in Ghana and Kenya. In both countries, there was no statistical difference in availability of QAACT with AMFm logo between RAs and nRAs in public health facilities (PHFs), while private-for-profit (PFP) outlets had lower availability in RA than in nRAs (Ghana: 66.0 vs 82.2 %, *p* < 0.0001; Kenya: 44.9 vs 63.5 %, *p* = <0.0001. The median price of QAACT with AMFm logo for PFP outlets in RAs (USD1.25 in Ghana and USD0.69 in Kenya) was above the recommended retail price in Ghana (US$0.95) and Kenya (US$0.46), and much higher than in nRAs for both countries. QAACT with AMFm logo represented the majority of QAACT in RAs and nRAs in Kenya and Ghana. In the PFP sector in Ghana, the market share for QAACT with AMFm logo was significantly higher in RAs than in nRAs (75.6 vs 51.4 %, *p* < 0.0001). In contrast, in similar outlets in Kenya, the market share of QAACT with AMFm logo was significantly lower in RAs than in nRAs (39.4 vs 65.1 %*, p* < 0.0001).

**Conclusion:**

The findings indicate the AMFm programme contributed to making QAACT more available in RAs in these two countries. Therefore, the AMFm approach can inform other health interventions aiming at reaching hard-to-reach populations, particularly in the context of universal access to health interventions. However, further examination of the factors accounting for the deep penetration of the AMFm programme into RAs is needed to inform actions to improve the healthcare delivery system, particularly in RAs.

## Background

Malaria burden remains high in sub-Saharan Africa and among various reasons for the sustained high burden of the disease in the region is the low uptake of the key malaria control interventions, including prompt treatment with recommended anti-malarial medicines for all the population in need, due in part to high cost of drugs [1]. The Global Fund to Fight AIDS, Tuberculosis and Malaria (Global Fund) hosted a Pilot Phase of the Affordable Medicines Facility-malaria (AMFm) in 2008 to increase uptake of effective anti-malarial medicines [2, 3]. AMFm is a financing mechanism consisting of: (1) price reductions through negotiations with manufacturers of quality-assured artemisinin-based combination therapy (QAACT); (2) Global Fund subsidy to buyers, through a co-payment to participating manufacturers for purchases made by eligible public, private and non-governmental organization importers; and, (3) interventions to support AMFm implementation and promote appropriate anti-malarial use [2, 3]. Under the AMFm mechanism, approved public and private importers or first-line buyers buy ACT from manufacturers at the subsidized price ranging from US$0.005 to US$0.220 for a treatment course, and then distributed them through the standard public and private sector distribution channels [4].

In 2010, the Pilot Phase of the AMFm was launched in eight national level programmes in seven countries in eastern Africa (Kenya, Uganda and the United Republic of Tanzania), western Africa (Ghana, Niger and Nigeria), and southern Africa (Madagascar) [2]. About 155.8 million doses of QAACT financed through AMFm were delivered to participating countries from August 2010 to December 2011 [5]. In addition to the medicines supplied to countries, several supporting interventions were implemented, including population awareness campaigns, setting recommended retail prices and training providers [2] to ensure effective implementation of the intervention.

The Global Fund commissioned an independent evaluation (IE) of AMFm to assess the achievement of its four objectives of reducing QAACT prices and increasing QAACT availability, market share and use at the national level in each pilot [5]. The IE used a pre- and post-test design and documented the implementation process and context in each pilot independently. National outlet survey of outlets stocking anti-malarial medicines was conducted at the baseline (2009/10) and the endline (2011) in each pilot. While assessing the performance of the programme at the national level, the Global Fund was also interested in knowing if the intervention had reached disadvantaged groups, particularly people living in areas considered remote. For this reason, the Global Fund commissioned a remote area study as part of the IE.

Traditionally, areas are classified as urban and rural with the latter often considered remote. However, some urban areas may be more remote than other urban areas, while some rural areas are more remote than other rural areas. Some rural areas that are better connected than isolated urban areas in some countries. To account for this potential misclassification, for in this study remoteness was defined as a function of distance from population settlements to service centres.

The remote area study involved a supplementary sample of remote areas in the AMFm outlet survey at endline. It was carried out in two of the pilot phase countries considered fast moving in implementing the AMFm intervention. Choosing fast-moving countries would allow sufficient implementation intensity and time for the intervention to have plausibly reached remote areas and, therefore, for an assessment to be informative. Fast-moving countries were expected to have received the co-paid medicines, started the intervention and been implementing the supporting interventions for about 12 months before the endline survey. At the time of selecting countries for the study, from all indications, Kenya and Ghana were fulfilling these criteria. This expectation was borne out by the main evaluation results, which found that Ghana and Kenya were among those that achieved several AMFm benchmarks.

In Ghana, the availability of QAACT increased by 52 % points from baseline to endline, meeting largely the benchmark 1 of a 20 % point increase in QAACT availability. Similarly, the country met the benchmark 5 of 10 % point increase in market share of QAACT with an increase of 40 % points of QAACT market share from baseline to endline. The median price of QAACT with AMFm was three times less than the median price of the most popular anti-malarial, which is not a QAACT in tablet form, just missing the benchmark 2 less than three times.

Kenya met, largely, the benchmark 1 with an increase of 35 % points of availability of QAACT from baseline to endline. Market share of QAACT increased significantly by 31 % point, meeting the benchmark 5. Compared to Ghana, Kenya met the benchmark 2 (price) with the median price of QAACT with AMFm equal to the median price of the most popular anti-malarial, which is not a QAACT in tablet form [4, 5].

However, the question remained of whether the AMFm programme benefitted more remote populations. This paper examines the availability, price and market share of QAACT in remote areas compared with non-remote areas in Kenya and Ghana at the endline of the AMFm intervention.

## Methods

### Study design and defining remoteness

The study was based on a non-experimental design comparing remote areas with non-remote areas using cross-sectional data collected from drug outlets in Kenya and Ghana. To define remoteness, the authors used weighted spatial access to different types of services centres as suggested by the Australian Institute for Health and Welfare [6, 7]. The authors generated a surface of travel time to service centres for Kenya and Ghana to define access to these centres and determine the degree of remoteness on a continuous surface of 1 × 1 km spatial resolution. Access to three layers of service centres was determined by assuming that people travel to a destination: (a) by walking or using non-motorized transport (cycling); (b) by walking from the place of residence through the landscape to the nearest road before finishing the remainder of the journey by motorized transport; or, (c) walking only from the origin along the road.

Population settlements were classified by distance to three service centres: Service Centres 1 (market and trading centres for Kenya; all grid squares with a population of 5000–10,000 for Ghana); Service Centres 2 (divisional headquarters and towns for Kenya; all grid squares with 10,000–50,000 population for Ghana); and Service Centres 3 (cities, municipalities, major towns and district headquarters for Kenya; all grid squares with a population of at least 50,000 for Ghana). Note, that for Kenya the authors used predefined settlement classifications by the Ministry of Roads and Public Works that mapped settlements in cities, municipalities, major towns, district headquarters, divisional headquarters, towns, market centres, and trading centres. For Ghana, because data were not readily available, the authors used the gridded population surface for 2010 at a resolution of 1 × 1 km [8] to define the service centres. It should be noted that we do not intend to compare the two countries, rather treat each country independently. Therefore, the difference in definition of remote areas between the two countries will not affect the analysis and interpretation of results.

The average travel time to any category of service centres was calculated from the 1 × 1 km grid surfaces for the two countries. The time it takes to travel to any category of service centres was divided by the average travel time to that category from each grid pixel. The result was a surface of travel time ratio-to-mean. A grid pixel with a ratio-to-mean travel time of 2 to Service Centre 1 implied it took twice as long to reach the nearest Service Centre 1 as it took from the average grid pixel. This ratio for each pixel was capped at a value of 0.5 for Kenya and 0.6 for Ghana to be equivalent to approximately half-an-hour travel time to a Service Centre 1, 1.5 h to a Service Centre 2 and 2 h to a Service Centre 3. All pixels with the ratio-to-mean travel time to any service centre of ≥0.5 were assigned ratio-to-mean of 0.5 [9] to reduce the effect of the longer travel times to larger but fewer service centres on the overall index. The sum of the capped ratio-to-mean surfaces to each type of Service Centre resulted in a continuous index of remoteness ranging from 0 to 1.5. The continuous surface was classified into five categories: highly accessible (≤0.3), accessible (>0.3 and ≤0.6), moderately accessible (>0.6 and ≤0.9), remote (>0.9 and ≤1.2), and very remote (>1.2 to 1.5) [7], then collapsed into two categories: non-remote (≤0.90) and remote areas (>0.90).

### Selecting remote area clusters

The IE outlet surveys at baseline and endline used a cluster sampling approach with urban and rural defined as domains. Clusters with on average 10,000–15,000 inhabitants, were selected randomly with probability proportional to population size [4]. Both baseline and endline surveys were powered to generate nationally representative estimates in rural and urban domains only; therefore, they did not include a sufficient number of clusters in remote areas to examine outcomes among this type of area. Using the final remoteness classification, the authors first identified the number of clusters from the IE endline outlet survey sample that were located in remote areas, and then estimated the number of additional clusters needed in remote areas to have 80 % statistical power and a 5 % significance level to detect differences in availability of co-paid QAACT between remote areas and non-remote areas, if any [5].

**Fig. 1:**
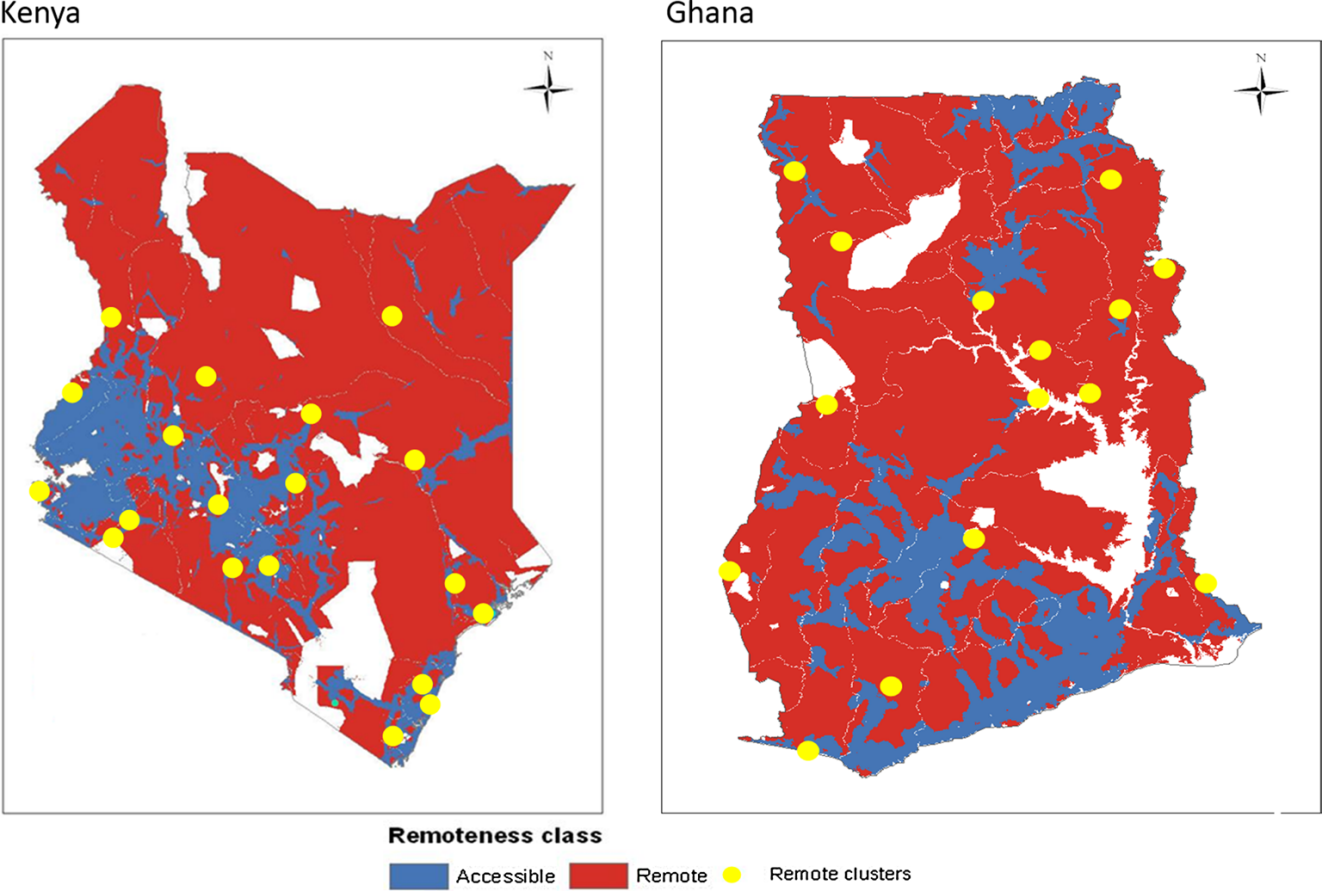
Remoteness classification map showing the location of clusters sampled in the remote areas for the AMFm Independent Evaluation remote area survey, 2012

In Kenya, 19 remote area clusters were required: the nine clusters identified in the IE endline outlet survey and ten additional clusters selected by probability proportional to population size. A total of 15 clusters were needed in Ghana: the five clusters identified in the IE endline outlet survey and ten additional clusters selected by probability proportional to population size. The spatial distribution of the clusters is depicted in Fig. 1. Note that for Kenya, non-malaria zones were excluded from the sampling frame used to select extra remote areas clusters. Furthermore the booster sample^1^ which had been included in the IE endline outlet survey was not collected in the new remote area clusters [5], because eligible facilities, Part One pharmacies and public health facilities were rare in the remote areas.

### Data collection

The surveys in the additional remote areas clusters were conducted from 27 February to 16 March, 2012 in Kenya and 4–13 March, 2012, in Ghana. Data were collected using paper-based questionnaires. The tools and procedures used in both countries were similar to those used in the IE endline outlet survey described in detail elsewhere [5]. In each selected cluster in remote areas, all outlets with the potential to sell anti-malarial medicines were enumerated. At each outlet, screening questions were asked and outlets that had anti-malarial medicines in stock or had stocked them in the previous 3 months were eligible for the full interview. After oral informed consent from the provider, a comprehensive questionnaire was administered. The questionnaire included provider characteristics and information on each anti-malarial in stock, including its type and retail price. The remote area survey took place after the end of the IE endline outlet survey. Therefore, a new question (“Have you stocked any anti-malarial medicines in the last 4 months? (November 2011–February 2012”) was added to the questionnaire to cover a longer recall period of 4 months to overlap with the IE endline outlet survey period October–December 2011 in both countries [5]. No new data were collected in the clusters in non-remote areas or remote areas that were covered in the IE endline outlet survey.

### Data processing and analysis

Data were entered using Microsoft Access in Kenya and Epidata in Ghana. Double entry and preliminary data cleaning were performed and data transferred to STATA v 11 for final cleaning, preparation of data and analysis. All anti-malarial medicines audited were classified into non-artemisinin therapy, artemisinin monotherapy (AMT), and artemisinin combination therapy (ACT). ACT included QAACT, with or without the AMFm logo, and non-QAACT. The QAACT with the AMFm logo were the co-paid medicines [5]. This paper reports anti-malarial medicine price and market share in adult equivalent treatment doses (AETDs), defined as the amount needed to treat a 60-kg adult. Market share was calculated by dividing the number of AETDs of a specific anti-malarial medicine category sold by the total number of AETDs of all anti-malarial medicines sold. Price data were collected in country currencies and adjusted to 2010 prices using national consumer price indices, then converted to US$ using the average interbank exchange rate for 2010 [4].

The analysis compares remote areas with non-remote areas in terms of availability, price and market share of QAACT in each country. Since booster sample was not included in the remote area survey, it was also removed from the non-remote areas before analysis. The significance of the differences between remote areas and non-remote areas was assessed using Chi squared tests for proportions and Wilcoxon rank sum tests for price indicators, expressed as medians. All the estimates in Kenya were weighted to account for the complex survey design. In Ghana, the estimates were not weighted because it was not possible to calculate the weight due to a lack of an estimate of the remote areas’ share of the total population [5]. Possible implications for the estimates are mentioned in the discussion.

### Ethical approval

Ethical approval was obtained from national ethics committees in Ghana and Kenya, and Institutional Review Boards of ICF International and the London School of Hygiene and Tropical Medicine.

## Results

### Description of the study sample

In Ghana, for the additional remote area survey, interviews were conducted in all 129 outlets that met the screening criteria, of which 117 were stocking anti-malarial medicines at the time of the survey visit. From the remote area clusters identified in the IE endline outlet survey, 32 outlets were stocking anti-malarial medicines. Therefore, 149 remote area outlets were included in the analysis. For the non-remotes, 487 outlets from the IE endline outlet survey that were stocking anti-malarial medicines were included in the analysis.

In Kenya, in the additional remote area survey, a much larger number of outlets was enumerated overall (3048) because in contrast to the case of Ghana, a broader grouping of outlets was classified as having the potential to sell anti-malarial medicines and thus were enumerated. For example, general retailers were systematically enumerated in Kenya but not Ghana. Interviews were conducted in 318 outlets, and 271 were stocking anti-malarial medicines at the time of the survey visit. From the remote area clusters identified in the IE endline outlet survey, 125 outlets were stocking anti-malarial medicines. The total sample for remote areas was therefore 396 outlets. For non-remote areas, 1223 outlets from the IE endline outlet survey were stocking anti-malarial medicines (Table 1).

**Table 1:** Number of remote and non-remote area outlets enumerated and number stocking antimalarials at the time of the survey in Ghana and Kenya, 2011–2012

Country/period of data collection	Outlets enumerated	Outlets screened	Outlets which met screening criteria	Outlets in which inter-views were conducted	Outlets stocking antimalarials at the time of the survey visit
Ghana					
Remote areas, total	221	194	164	164	149
In additional survey^a^	161	147	129	129	117
In endline survey^b^	60	47	35	35	32
Non-remote areas only in endline survey	–	–	506	506	487
Kenya					
Remote areas, total	4244	3241	468	468	396
In additional survey^a^	3048	2353	318	318	271
In endline survey^b^	1196	888	150	150	125
Non-remote areas only in endline survey	–	–	9980	9980	1223

^a^Data collection period: Ghana: November 7–28, 2011, Kenya: October 7–December 10, 2011

^b^Data collection period: Ghana: March 4–13, 2012, Kenya: February 27–March 16, 2012

Of the 149 outlets with anti-malarial medicines in Ghana, almost two-thirds (97) were private for-profit outlets and about one-sixth (26) were public health facilities. A similar pattern was observed in Kenya, where private for-profit outlets represented 82.8 % (328) of all outlets with anti-malarial medicines, followed by public health facilities. In contrast to Ghana, where 22 community health workers had anti-malarial medicines in stock, in Kenya only one community health workers reported having anti-malarial medicines in stock (Table 2).

**Table 2:** Number of outlets with antimalarials in stock by type of outlet in the remote and non-remote areas in Ghana and Kenya, 2011–2012

Country/type of outlets	Remote areas	Non-remote areas
Ghana, total	149	487
Public health facility	26	55
Private not-for-profit health facility	4	10
Private for-profit outlet	97	422
Community health worker	22	0
Kenya, total	396	1223
Public health facility	52	105
Private not-for-profit health facility	15	43
Private for-profit outlet	328	1075
Community health worker	1	0

### Availability of quality-assured artemisinin combination therapy

Public health facilities in both countries had statistically similar levels of availability in remote areas and non-remote areas (Ghana: 96.2 vs 80.0 %, *p* = 0.0645 and Kenya: 95.4 vs 96.4 %, *p* = 0.9999). Private for-profit outlets presented a different picture, with significantly lower availability of QAACT in remote areas in both countries (Ghana: 68.0 vs 84.1 %, *p* < 0.0001 and Kenya: 45.9 vs 65.5 %, *p* < 0.0001). In both countries, most outlets in all areas had QAACT in stock. However, in Ghana there was no significant difference in availability of QAACT in all types of outlets combined between remote areas and non-remote areas. In Kenya, the availability of QAACT in all types of outlets combined was significantly lower in remote areas than in non-remote areas (56.2 vs 70.8 %, *p* < 0.0001).

In public health facilities in both countries, there was no statistical difference in availability of QAACT with the AMFm logo between remote areas and non-remote areas (Ghana: 84.6 vs 76.4 *%, p* = 0.3951 Kenya: 60.5 vs 68.9 %, *p* = 0.9913). In both countries, private for-profit outlets had lower availability of QAACT with the AMFm logo in remote areas than in non-remote areas (Ghana: 66.0 vs 82.2 %, *p* < 0.0001; Kenya: 44.9 vs 63.5 %, *p* = <0.0001). The availability of QAACT with the AMFm logo was significantly lower in all types of outlets combined in remote areas than in non-remote areas in both countries. Nevertheless, in remote areas almost half the outlets in Ghana and 60 % in Kenya had QAACT with the AMFm logo. It should be noted that availability of all QAACT is very similar to QAACT with AMFm logo as most of QAACT in private for-profit outlets were AMFm-subsidized QAACT (Table 3).

**Table 3:** Outlets in remote areas and non-remote areas with quality-assured artemisinin combination therapies in stock at endline in Ghana and Kenya, 2011–2012

Country/Type of outlet	Remote areas		Non-remote areas		p value*
	Percentage (95 % CI)	N	Percentage (95 % CI)	N	
All QAACTs					
Ghana, total	77.9 (67.0–85.9)	149	83.8 (78.8–87.8)	487	0.1634
Public health facility	96.2 (74.5–99.5)	26	80.0 (63.6–90.1)	55	0.0645
Private not-for-profit health facility^a^	100.0	4	90.0 (57.5–98.4)	10	–
Private for-profit outlet	68.0 (47.6–83.3)	97	84.1 (78.5–88.5)	422	<0.0001
Community health worker^a^	95.5 (80.0–99.1)	22	–	0	–
Kenya, total	56.2 (43.4–68.2)	392	70.8 (63.8–76.8)	1223	<0.0001
Public health facility	95.4 (82.2–98.9)	51	96.4 (89.9–98.8)	105	0.9999
Private not-for-profit health facility^a^	100.0	15	98.6 (93.1–99.7)	43	0.561
Private for-profit outlet	45.9 (30.0–62.6)	325	65.5 (57.2–72.9)	1075	<0.0001
Community health worker^a^	100.0	1	–	0	–
QAACTs with AMFm Logo					
Ghana, total	60.4 (37.7–79.3)	149	81.5 (76.7–85.6)	487	<0.0001
Public health facility	84.6 (65.6–94.1)	26	76.4 (59.4–87.7)	55	0.3951
Private not-for-profit health facility^a^	100.0	4	80.0 (49.4–94.2)	10	–
Private for-profit outlet	66.0 (45.9–81.6)	97	82.2 (76.9–86.5)	422	<0.0001
Community health worker^a^	0.0	22		0	–
Kenya, total	48.5 (36.2–61.0)	392	64.0 (56.3–71.0)	1223	<0.0001
Public health facility	60.5 (44.6–74.4)	51	68.9 (54.0–80.8)	105	0.9913
Private not-for-profit health facility	73.3 (44.0–90.6)	15	58.8 (37.0–77.6)	43	0.3622
Private for-profit outlet	44.9 (29.2–61.8)	325	63.5 (55.6–70.8)	1075	<0.0001
Community health worker	100.0	1	–	0	–

Percentages indicate the percentage of outlets in remote areas and non-remote areas that had quality-assured ACTs in stock at the time of the survey visit (n) among all outlets with any antimalarial in stock at the time of the survey visit (N), by type of outlet, according to country, 2011–2012. CI: Confidence interval; AMFm = Affordable Medicine Facility-malaria

* p value for Chi square testing for difference between remote and non-remote areas

^a^These are presented in the table because they contribute to the country overall estimates; however, they are not discussed in the text because of the small sample

### Pricing of quality-assured artemisinin combination therapy

In Ghana, public health facilities and private for-profit outlets, the median cost to patients of one AETD of all QAACT in all formulations (adult and children) was not significantly different between remote areas and non-remote areas (public health facilities: US$0.94 vs US$0.94, *p* = 0.3742; private for profit outlets: US$1.25 vs US$1.25, *p* = 0.9742). In Kenya, the median price of QAACT was zero in public health facilities that are required to provide ACT for free, in remote areas and non-remote areas. However, the private for-profit outlets in remote areas were selling QAACT at nearly twice the price as in non-remote areas (US$0.81 vs US$0.46, *p* < 0.0001). Regarding QAACT with the AMFm logo, in both countries the median price for PHFs was similar in remote areas and non-remote areas. However, in Kenya the median price of QAACT with the AMFm logo was much higher in remote areas for private for-profit outlets than in non-remote areas (US$0.69 vs US$0.46, *p* < 0.0001). Similarly in Ghana, private for-profit in remote areas were selling QAACT with AMFm logo US$0.25 higher than non-remote areas (Table 4).

**Table 4:** Median cost to patients of one adult equivalent treatment dose of all formulations (adults and children) of quality-assured artemisinin combination therapies in remote areas and non-remote areas at endline in Ghana and Kenya in US dollars, 2011–2012

Country/type of outlet	Remote areas		Non-remote areas		p value*
	Median cost (IQR)	N	Median cost (IQR)	N	
All QAACTs					
Ghana, total	1.25 (0.94–1.88)	187	0.95 (0.94–1.88)	923	0.0652
Public health facility	0.95 (0.94**–**1.88)	46	0.94 (0.94**–**0.94)	66	0.3742
Private not-for-profit health facility^a^	0.94 (0.00**–**0.94)	5	0.94 (0.00**–**0.94)	20	0.2113
Private for-profit outlet	1.25 (0.94**–**1.88)	114	1.25 (0.94**–**1.88)	837	0.9742
Community health worker^a^	1.25 (1.25**–**1.50)	22	–	0	–
Kenya, total	0.00 (0.00–0.69)	412	0.46 (0.00–0.61)	1864	<0.0001
Public health facility	0.00 (0.00**–**0.00)	182	0.00 (0.00**–**0.00)	342	–
Private not-for-profit health facility	0.00 (0.00**–**0.31)	46	0.00 (0.00**–**1.04)	116	0.2534
Private for-profit outlet	0.81 (0.46**–**1.38)	180	0.46 (0.46**–**0.92)	1406	<0.0001
Community health worker^a^	1.73 (1.15**–**3.45)	4	–	0	–
QAACTs with AMFm logo					
Ghana, total	1.00 (0.94–1.88)	156	0.94 (0.94–1.88)	845	0.1121
Public health facility	0.94 (0.94**–**1.25)	39	0.94 (0.94**–**0.94)	62	0.0624
Private not-for-profit health facility^a^	0.94 (0.00**–**0.94)	5	0.94 (0.00**–**0.95)	19	0.1971
Private for-profit outlet	1.25 (0.94**–**1.88)	112	1.00 (0.94**–**1.88)	764	0.0363
Community health worker^a^	–	0	–	0	–
Kenya, total	0.46 (0.00–1.15)	292	0.46 (0.46–0.69)	1539	0.2423
Public health facility	0.00 (0.00**–**0.00)	90	0.00 (0.00**–**0.00)	156	–
Private not-for-profit health facility	0.00 (0.00**–**0.31)	26	0.46 (0.00**–**0.69)	45	0.2612
Private for-profit outlet	0.69 (0.46**–**1.38)	172	0.46 (0.46**–**0.92)	1338	<0.0001
Community health worker^a^	1.73 (1.15**–**3.45)	4	–	0	–

*IQR* interquartile range, *N* number of products audited, *AMFm* affordable medicine facility-malaria

* p value for Wilcoxon rank sum test for difference between remote and non-remote areas

^a^These are presented in the table because they contribute to the country overall estimates; however, they are not discussed in the text because of the small sample

The median price of a paediatric formulation^2^ of all QAACT for public health facilities and private for-profit outlets did not differ between remote areas and non-remote areas in Ghana and Kenya. Similarly, in both countries the median price paediatric formulation of QAACT with the AMFm logo in public health facilities and private for-profit outlets was the same in remote areas and non-remote areas (Table 5).

**Table 5:** Median cost to patients of a pediatric dose (unit-dose packages specifically intended to treat a 2 year/10 kg child) formulation of quality-assured artemisinin combination therapies in remote areas and non-remote areas at the endline in Ghana and Kenya, in US dollars, 2011**–**2012

Country/type of outlet	Remote areas		Non-remote areas		p value**
	Median cost (IQR)	N	Median cost (IQR)	N	
All QAACTs					
Ghana, total	0.38 (0.31–0.63)	50	0.63 (0.63–0.94)	156	0.0102
Public health facility	0.28 (0.24**–**0.47)	12	0.24 (0.24**–**0.47)	5	0.7121
Private not-for-profit health facility^a^	–	0	0.59 (0.24**–**0.94)	2	–
Private for-profit outlet	0.63 (0.63**–**0.94)	19	0.63 (0.63**–**0.94)	149	0.7731
Community health worker^a^	0.31 (0.31**–**0.38)	19	–	0	–
Kenya, Total	0.00 (0.00–0.46)	65	0.35 (0.00–0.46)	254	<0.0001
Public health facility	0.00 (0.00**–**0.00)	27	0.00 (0.00**–**0.00)	41	–
Private not-for-profit health facility^a^	0.00 (0.00**–**0.23)	10	0.00 (0.00**–**0.46)	14	0.8642
Private for-profit outlet	0.46 (0.35**–**0.58)	27	0.46 (0.35**–**0.46)	199	0.2123
Community health worker^a^	0.58	1	–	0	–
QAACTs with AMFm logo					
Ghana, total	0.63 (0.42–0.84)	24	0.63 (0.63–0.94)	147	0.4033
Public health facility	0.24 (0.24**–**0.47)	7	0.24 (0.24**–**0.43)	4	0.9041
Private not-for-profit health facility^a^	–	0	0.59 (0.24**–**0.94)	2	–
Private for-profit outlet	0.63 (0.63**–**0.94)	17	0.63 (0.63**–**0.94)	141	0.8613
Community health worker^a^	–	0	–	0	–
Kenya, total	0.00 (0.00–0.46)	52	0.35 (0.23–0.46)	212	<0.0001
Public health facility	0.00 (0.00**–**0.00)	21	0.00 (0.00**–**0.00)	19	0.4845
Private not-for-profit health facility^a^	0.00 (0.00**–**0.00)	4	0.00 (0.00**–**0.58)	6	0.7213
Private for-profit outlet	0.46 (0.35**–**0.58)	26	0.46 (0.35**–**0.46)	187	0.2731
Community health worker^a^	0.58	1	–	0	–

*IQR* interquartile range, *N* number of products, *AMFm* affordable medicine facility-malaria

** p value for Wilcoxon rank sum test for difference between remote and non-remote areas

^a^These are presented in the table because they contribute to the country overall estimates; however, they are not discussed in the text because of the small sample

### Market share for QAACT

**Fig. 2:**
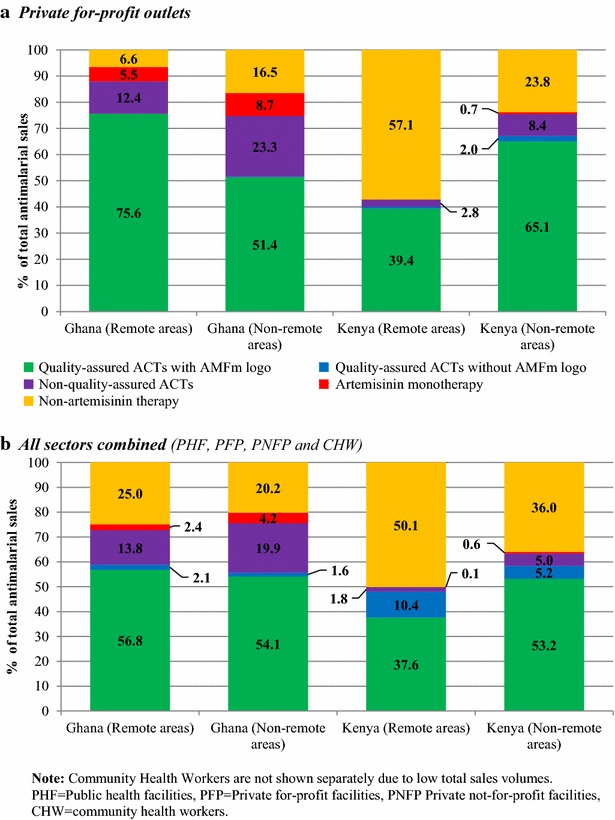
Market share of anti-malarials by anti-malarial type in remote and non-remote areas, 2011–2012

In the private for-profit PFP sector alone, in Ghana, QAACT had the dominant market share in remote areas and non-remote areas, but the market share for QAACT was significantly higher in remote areas than in non-remote areas (75.6 vs 51.5 %, *p* < 0.0001) and close to 100 % of the QAACT had the AMFm logo. In Kenya, in the private for-profit outlets, the market share of QAACT was significantly lower in remote areas than in non-remote areas (40.0 vs 67.0 %*, p* < 0.0001) and nearly all the QAACT had the AMFm logo (Fig. 2a). In Ghana, across all outlets, the market share of QAACT was similar in remote areas (58.8 %) and non-remote areas (55.7 %). Non-artemisinin therapy had the second highest market share in remote areas and non-remote areas (25.0 vs 20.2 %). In Kenya, the market share for QAACT was significantly lower in remote areas than in non-remote areas (48.0 vs 58.4 %, *p* < 0.0001). In both countries, QAACT with the AMFm logo represented the majority of QAACT in remote areas and non-remote areas (Fig. 2b).

## Discussion

The study compared the availability, price and market share of QAACT in remote areas and non-remote areas, defined as distance and travel time to major service centres, at the endline of the AMFm pilot phase in Ghana and Kenya. Although there had been considerable uncertainty about whether AMFm would reach remote areas, the results presented here confirm that remote areas benefitted from the intervention to a considerable extent.

Although the availability of QAACT was lower in remote areas than in non-remote areas, the medicines were widely available in remote areas in Ghana and Kenya at the endline. The availability of QAACT was particularly high in public health facilities; however, substantial private for profit outlets and most medicines were QAACT with the AMFm logo in both countries. Overall, the findings suggest that the AMFm programme was instrumental in making QAACT more available in remote areas in these countries. Yadav and colleagues reported similar finding in Tanzania where AMFm led to a large increase in the availability of QAACT with the AMFm logo in the country, with variation based on remoteness [10]. In Kenya and Ghana, reliable distribution systems, particularly in the private sector, could have played a major role in making the medicines available in remote areas. Ghana and Kenya are among the countries that met the AMFm benchmark indicator of a 20 % point increase in QAACT availability from the baseline to the endline [4].

The median price of QAACT (adult and children’s formulations) in remote areas and non-remote areas was similar in Ghana. On the other hand in Kenya, the median price of QAACT was US$0.46 in non-remote areas and almost zero in remote areas. The near-zero median price in remote areas in Kenya is driven by the substantial number of public health facilities where the drug is mostly free. In contrast, patients paid for QAACT in public health facilities in Ghana [5]. However, there was no difference in the median price of QAACT pediatric formulation in the public health facilities and private for-profit outlets between remote areas and non-remote areas in both countries. These findings are very comforting as for Ghana and Kenya children under-five are the most vulnerable to malaria infection.

At the inception of the AMFm, there were concerns the price of QAACT with AMFm logo will be higher than the recommended retail price in the private sector in remote areas due to potential challenges and additional cost for distribution, and limited competition. However, in Ghana and Kenya, the median retail price for QAACT with AMFm logo in private for profit outlets were in line with the recommended retail price (US$0.94) in Ghana and in Kenya (US$0.46). Despite the challenges and additional cost related to distribution of the medicines in remote areas, private sector provider mark-up was not excessive. These findings are in line with those by Yadav and colleagues in Tanzania mainland where they found no difference in price of QAACT with AMFm logo between remote areas and non-remote areas [9]. In Ghana and Kenya, several factors could have contributed to widespread compliance to the recommended retail price for co-paid QAACT; significant participation by the private sector in the AMFm implementation process in both countries is likely to have been a contributing factor.

In Ghana, although several anti-malarial medicines were on the market, for all sectors combined there was no difference between remote areas and non-remote areas in the market share of QAACT. However the market dominance of QAACT in the private for-profit sector, especially in remote areas, suggests that the increased availability and reduced price may have increased community awareness of QAACT, resulting in its higher level of (relative) sales. In contrast, in Kenya in the private for-profit outlets in remote areas, the market share was dominated by non-artemisinin therapy. The lower market share of QAACTs in the private for-profit outlets in remote areas is probably due to the higher price. Nevertheless, as in Ghana, Kenya achieved the AMFm market share benchmark of a 10 % point increase in market share of QAACT [4]. A possible explanation for the relatively low uptake of QAACT in RAs compared with nRAs in Kenya could be a lack of community awareness of the AMFm programme in remote areas.

There are a number of limitations that should be considered when using the findings. Remoteness was defined as distance and travel time to service centres. The assumptions underlying the travel times assigned to different land surfaces may vary by country-specific context and may therefore, overestimate or underestimate remoteness. Secondly, the outlet survey relied on self-reports of sales volume and prices with potential recall or reporting bias. The authors tried to minimize the recall bias by asking for reported sales volumes only for the week preceding the survey; however, recall may still have been imperfect [11]. Thirdly, the additional remote area outlet survey was conducted in the low malaria transmission season, and non-remote area data collected during a higher transmission season in both countries, therefore, some of the differences between remote areas and non-remote areas might be due to differences in data collection dates. Fourthly, the comparison of remote areas versus non-remote areas was only undertaken for Ghana and Kenya, which were the best-performing countries overall in AMFm; their experience in remote areas may have been different in countries with poorer AMFm outcomes. Finally, the lack of weights for Ghana may have created bias in the comparison between remote areas and non-remote areas in that country, as most of the larger outlets most likely to have QAACT were probably in non-remote areas.

## Conclusion

Despite the challenges in geographical access posed by remote areas and the relatively short intervention period, the results show that the AMFm intervention has been able to reach remote areas in Ghana and Kenya. Although this cross-sectional evidence does not permit strong inference about changes over time, it is likely that the availability of QAACT increased in remote areas, as the majority of QAACT were those with the AMFm logo, not available at IE baseline. However, even though co-paid medicines was available in remote areas in the private for profit sector, availability remained lower, price higher, and (in Kenya) market share was lower than in non-remote areas.

The health care delivery system could learn from this success and the challenges in the private sector, and could adapt a similar approach to adequately cover remote and hard-to-reach populations with other interventions. However, further examination of the factors accounting for the deep penetration of the AMFm programme into remote areas is needed to inform actions to improve the health sector commodities distribution systems.

## Footnotes

1 Public health facilities and Part one pharmacies typically serve a large number of patients and may be the main providers of QAACT. However, few of these outlets were expected to be in any given cluster. Therefore, public health facilities and part one pharmacies were oversampled. For each sampled subdistrict, public health facilities and part one pharmacies found in the district in which the sub-district was located were censused [5], AMFm Independent Evaluation Team 2012).

2 Unit-dose packages intended for a 2-year old/10-kg child.
